# Effects of dietary intake patterns from 1 to 4 years on BMI *z*-score and body shape at age of 6 years: a prospective birth cohort study from Brazil

**DOI:** 10.1007/s00394-018-1720-3

**Published:** 2018-05-17

**Authors:** Leonardo Pozza Santos, Ken K. Ong, Ina S. Santos, Alicia Matijasevich, Aluísio J. D. Barros

**Affiliations:** 1 Post-graduate Program in Epidemiology, Federal University of Pelotas, Pelotas, Brazil; 2 Nutrition School, Federal University of Pampa, Luiz Joaquim de Sá Britto, s/n, Itaqui, 97650-000 Brazil; 3 Medical Research Council (MRC) Epidemiology Unit, Institute of Metabolic Science, Cambridge, UK; 4 Department of Preventive Medicine, Faculty of Medicine FMUSP, University of São Paulo, São Paulo, Brazil

**Keywords:** Diet, Food intake, Body mass index, Body shape

## Abstract

**Purpose:**

To assess the association between dietary intake patterns from 1 to 4 years and BMI and body shape at age of 6 years.

**Methods:**

This longitudinal study was based on 3374 Brazilian children from the 2004 Pelotas Birth Cohort Study. We used previously described dietary patterns from 1 to 4 years as the main exposure. We defined body shape using scores for *corpulence* (a recently described body shape component measured by Photonic Scanner), and trunk and gynoid fat mass percentage from DXA. We run linear regression models to evaluate the associations between dietary patterns from 1 to 4 years and BMI and body shape at 6 years.

**Results:**

Several apparent associations between dietary patterns and BMI or body shape were explained by sociodemographic factors. High adherence to *snacks* (positive loadings to coffee, bread and cookies) at 4 years predicted lower BMI, but higher gynoid fat mass percentage at 6 years, while higher adherence to *staple* at 2 years (positive loadings to rice and beans) predicted higher trunk fat mass and lower gynoid fat mass. Finally, higher scores on *milks* at 1 year (positive loading to breast milk) predicted higher gynoid fat mass at 6 years.

**Conclusion:**

There were inconsistent associations between dietary patterns in infancy and early childhood and BMI and body shape at 6 years. In adjusted analyses, higher adherence to breast milk at 1 year and to *snacks* at 4 years appeared to be beneficial for body shape, associated with lower BMI, but higher peripheral fat.

**Electronic supplementary material:**

The online version of this article (10.1007/s00394-018-1720-3) contains supplementary material, which is available to authorized users.

## Introduction

Latin American countries faced nutrition transition in the recent years, where a ‘traditional’ diet has been replaced by a more ‘Westernized’ diet, impacting on obesity rates [1–3]. Recent estimates showed that, in Latin America, more than 20 million of children aged 0–19 years are overweight or obese, representing 20–25% of the overall children population [4]. Specifically in Brazil, more than 1/3 of children are overweight or obese according to recent estimates [5].

Obesity status may be affected by the type of food consumed in childhood as well as by the interaction among food components [6, 7]. However, it is a challenge measuring food and nutrient intake in childhood, and principal component analysis (PCA) has emerged as a useful tool to assess dietary intake patterns, summarizing predominant models of intake [8, 9]. Some evidences have suggested that dietary intake patterns are stable since the early ages [10–12], but the effect of dietary intake patterns on obesity status and adiposity is quite inconsistent [13–15].

The majority of papers assessing the association between dietary intake patterns and adiposity has been conducted in high-income countries and has used body mass index (BMI) as the main outcome. As BMI has inherent limitations, such as do not distinguish fat mass from fat-free mass and do not allow to assess body fat distribution, new alternatives to measure body composition and body shape have arisen in last years [16, 17]. More recently, the three-dimensional photonic scanner (3DPS) has been used as a new alternative to measure body shape and size in different populations. This equipment projects lights on the body surface and read it back, and computer algorithms create a 3D body image of the surface. A specialized software uses this 3D image to calculate several body measures such as girths, lengths, areas, and volumes. When measuring waist circumference, the 3DPS-generated measures were greater, but showed high correlation and ranking consistency in children and adults when compared to tape-based measures [18, 19]. This approach was used in the 6-year-old follow-up of the 2004 Pelotas Birth Cohort Study to describe the components of body shape and size of children [20].

We have previously identified and described dietary intake patterns in infancy (1 and 2 years) and early childhood (4 years) [21], and also body shape and size at 6 years. Here, we describe the relationship between those early life dietary intake patterns and BMI *z*-score and body shape at 6 years in this population-based sample from Brazil.

## Methods

### Subjects

Pelotas is a municipality with around 340,000 inhabitants situated in southern Brazil. Currently, four birth cohort studies are being followed up at regular intervals (the 1982, the 1993, the 2004, and the 2015 Pelotas Birth Cohort Studies), including newborns from mothers residing in the city’s urban area. Specifically for this study, we used data from the 2004 Pelotas Birth Cohort Study, since information about dietary intake patterns from 1 to 4 years of age as well as body shape components at 6 years are only available for this cohort.

In 2004, 99.2% (4231) of all babies born from mothers living in the urban area were recruited to take part of the 2004 Pelotas Birth Cohort Study. In the first 24 h after birth, the interviewers recruited and interviewed mothers, and evaluated the newborns at maternity hospitals. They collected information about family, maternal characteristics, the current pregnancy, and delivery. Children were also followed at ages of 3 months, and 1, 2, 4, and 6 years, with follow-up rates of 95.7, 94.2, 93.4, 91.8, and 90.2%, respectively. Details of all follow-ups were reported previously [22, 23]. The Research Ethic Committee of the Medical School from the Federal University of Pelotas approved all follow-up waves of the study; each time, the children’s legal guardians gave their written informed consent to participate in the study.

### Dietary intake

Children’s dietary intake during the 24-h prior to interviews at ages 1, 2, and 4 years was assessed by a questionnaire completed by the child’s mother. The questionnaires used in 1-, 2-, and 4-year-old follow-ups asked mothers to report whether each food item from a list had been consumed during meals or periods of the day (breakfast, morning, lunch, afternoon, dinner, evening, and night), but they do not allow to quantify the amount of foods consumed at each period of the day. This list of commonly consumed food items was constructed based on children’s food intake collected in a multicentre study conducted in Pelotas (WHO Multicentre Growth Reference Study in Brazil) [24].

Dietary intake patterns at 1, 2, and 4 years were identified by PCA and previously described by Gatica et al. [21]. Each dietary pattern comprises food items with positive or negative loadings. Five components were identified at each age; four of them similar in all time points, as described below.

Five dietary patterns were identified at ages 1 and 2 years:

- *Milks* (positive loading for breast milk; negative loading for cow’s milk);
- *Staple* (positive loading for rice and beans; negative loading for pasta);
- *Beverages* (positive loading for juice; negative loading for water and tea);
- *Snacks* [positive loading for coffee, bread and cookies; negative loading for fruits (1 year) or yogurt (2 years)];
- *Meat and vegetables* [positive loading for meats, vegetable and legumes, potato and cassava, and fruits (only 2 years)].

At age 4 years, child diets comprised five slightly different patterns:

- *Milks* (positive loading for cow’s milk and chocolate powder);
- *Staple* (positive loading for rice, beans, and meat);
- *Beverages* (positive loading for juice; negative loading for soft drinks);
- *Snacks* (positive loading for coffee, bread and cookies, and water and tea; negative loading for yogurt and soft drinks);
- *Treats* (positive loading for crisps, sweets, and chocolate).

Supplementary table 1 shows details about dietary intake patterns at the three age-points, including all food items with positive and negative loadings of each component.

### Body mass index and body shape

The 6-year-old follow-up of the 2004 Pelotas birth cohort study occurred between 2010 and 2011 and followed up 3722 children. In this follow-up, weight was measured by a high precision scale (0.01 kg) linked to BodPod machine (Cosmed, Italy, http://goo.gl/7jzfLc). Height was measured twice by trained field workers using a Harpenden metal stadiometer, with 1-mm precision (Holtain, Crymych, UK). BMI was then calculated by dividing weight (kg) by height (m) squared and standardized by age and sex using the World Health Organization (WHO) 2007 growth reference [25] for 3374 children who had available information about weight and height. The field workers who conducted anthropometric measures were standardized according to the Habitch criteria [26].

Body shape was assessed using TC^2^ Three-Dimensional Photonic Scanner (North Carolina, USA; http://www.tc2.com). Two photonic scans were performed in each child, capturing 38 measurements among circumferences, lengths, volumes, and surface areas. If the difference in waist circumference was greater than 10 mm, a third scan was performed, and arithmetic mean of those two measurements with difference lower than 10 mm was calculated. Specially trained field workers carried out all photonic scans and the machine was calibrated in the beginning of each working day.

Particularly relevant for our study, we defined body shape according to the components of body shape and size at 6 years described by Santos et al. [20]. We used the major component called *corpulence*, which explained almost 70% of the variance in children’s body shape and size, and was the only one which was correlated with the traditional anthropometric (weight, BMI, waist circumference, and height) and body composition measures (fat, lean and bone mass, and trunk, android, and gynoid fat mass) (Pearson’s correlation > 0.70). Briefly, *corpulence* included positive loadings for measures of body circumferences (waist, hip, seat, chest, abdomen, knee, calf, and biceps circumferences), diameters (sagittal diameter, waist, and abdomen width), and volumes (body volume and torso volume) [20].

As *corpulence* is a relatively novel approach to define body shape, we also used central and peripheral fat mass measures as additional body shape definitions, to facilitate interpretation of the findings. We assessed regional fat mass using dual-energy X-ray absorptiometry (DXA) (GE Lunar Prodigy densitometer). Specific trained field workers collected DXA scans following the manufacturer’s recommendations and position protocols. We used trunk and gynoid fat mass percentage from DXA as measures of central and peripheral body shape, respectively. Trunk and gynoid fat mass percentage were defined as follows:

$$\begin{aligned} {\text{Trunk fat mass percentage }}= & {\text{ }}\left( {{\text{trunk fat mass }}\left( {{\text{kg}}} \right)/{\text{total fat mass }}\left( {{\text{kg}}} \right)} \right]~ \times ~{\text{1}}00. \\ {\text{Gynoid fat mass percentage }}= & {\text{ }}\left[ {{\text{gynoid fat mass }}\left( {{\text{kg}}} \right)/{\text{total fat mass }}\left( {{\text{kg}}} \right)} \right]~ \times ~{\text{1}}00. \\ \end{aligned}$$

### Socioeconomic and demographic information

Socioeconomic position (SEP) was measured according to the Brazilian National Wealth Index (IEN). Briefly, this index considers household assets and the household head’s education according to Brazilian Demographic Census data [27]. We also used information about maternal schooling (years of formal education), number of children at the time of birth, maternal age at birth, maternal smoking during pregnancy, child’s sex and skin colour, birth weight, exclusive breastfeeding duration, and number of meals per day at 1, 2, and 4 years of age. All the details about data collection of socioeconomic and demographic variables were reported previously [22].

### Statistical analysis

To analyze dietary patterns, we categorized the scores of each dietary intake component at 1, 2, and 4 years into tertiles, representing low (first tertile), intermediate (second tertile), and high scores (third tertile). Scores for *corpulence* were standardized providing a mean of 0 and standard deviation 1. Associations between dietary patterns at 1, 2, and 4 years and BMI *z*-score or *corpulence* at 6 years were assessed using multiple linear regression separately by each age-point.

We performed crude (Supplementary tables 2, 3) and adjusted analyses, considering as potential confounders those variables which were associated (*p* value ≤ 0.2) with at least one exposure and one outcome, to keep the same model for all the analyses tested in the study. All associations were adjusted for socioeconomic position, number of siblings, maternal age at birth and education, smoking during pregnancy, child’s sex and skin colour, birth weight, exclusive breastfeeding duration, and number of meals per day.

We conducted additional analyses using regional measures of body shape from DXA (trunk and gynoid fat mass percentage). As both BMI and *corpulence* were standardized, we also standardized values for trunk and gynoid fat mass percentage to keep the same unity for all outcomes. Furthermore, we performed here the same adjustment used for BMI and *corpulence*.

We evaluated dietary patterns separately by each age-point (1, 2, and 4 years), since their food item loadings varied slightly by age, and there were weak inter-correlations between components (Pearson’s correlation ≤ 0.50), making it difficult to combine components across ages. Nonetheless, dietary components at the previous age were always associated with similar-labeled components at next age (*p* value s < 0.20), despite weak correlations among them (unpublished results). Then, when the main exposure was dietary intake patterns at 2 and 4 years, we also adjusted for similar-labeled components at the previous age. For example, when the main exposure was *milks* at 2 years, we included in the model scores of *milks* at 1 year as a potential confounder. When the main exposure was *milks* at 4 years, we included scores of *milks* at 1 and 2 years in the model.

We evaluate whether sex modifies the effect of dietary intake patterns on BMI and body shape using the product term of child’s sex and each outcome. In all models, we assessed the variance inflation factor to assess for multicollinearity among independent variables. All analyses were carried out using Stata 13.1 (Stata Corp., College Station, TX, USA).

## Results

We included 3374 children (52% boys) in the analysis for whom information on BMI at 6 years was available. The mean weight was 25 kg (± 5 kg) and the mean height was 1.21 m (± 0.06). 35% of children were classified as overweight or obese according to the WHO 2007 international growth reference. Table 1 presents socioeconomic, maternal, and children’s characteristics. Mothers were more likely to have at least 5 years of formal education, at least two children, and were also more likely to age 18–35 years by the time of birth. Children were more likely to be boys and white, and the averaged birth weight of this sample was 3.2 kg (Table 1). The mean of daily meals decreased from 1 to 4 years (6.3 meals at 1 year, 5.9 at 2 years and 5.2 at 4 years), and adherence to dietary intake components had small effect on the number of meals per day (Supplementary table 4).

**Table 1:** Socioeconomic, maternal, and children’s characteristics

Characteristics	Followed-up, *N* (%)	Dropped, *N* (%)	*p* value
SEP (quintiles)			0.001
First (poorest)	763 (22.7)	262 (30.8)	
Second	718 (21.4)	146 (17.2)	
Third	763 (22.7)	157 (18.5)	
Fourth	555 (16.5)	112 (13.2)	
Fifth (richest)	561 (16.7)	174 (20.5)	
Maternal education (years)			0.002
0–4	505 (15.1)	150 (17.8)	
5–8	1403 (42.0)	328 (38.9)	
≥ 9	1436 (42.9)	366 (43.4)	
Number of children			0.005
1	1345 (39.9)	321 (37.5)	
2	895 (26.5)	216 (25.2)	
3	546 (16.2)	134 (15.6)	
≥ 4	587 (17.4)	186 (21.7)	
Maternal age at birth (years)			0.425
< 18	323 (9.6)	73 (8.5)	
18–35	2687 (79.7)	704 (82.2)	
> 35	363 (10.8)	79 (9.2)	
Maternal smoking at pregnancy			0.167
No smoking	2464 (74.9)	614 (73.3)	
< 20 cigarettes/day	788 (24.0)	214 (25.5)	
≥ 20 cigarettes/day	36 (1.1)	10 (1.2)	
Children’s sex			0.366
Boys	1739 (51.5)	193 (57.4)	
Girls	1635 (48.5)	143 (42.6)	
Children’s skin colour (mother’s report)			0.392
White	2249 (71.4)	235 (74.1)	
Brown	487 (15.5)	41 (12.9)	
Black	414 (13.1)	41 (12.9)	
Birth weight (g)			< 0.001
< 2500	289 (8.6)	120 (14.9)	
≥ 2500	3085 (91.4)	683 (85.1)	
Exclusive breastfeeding duration			0.054
≤ 7 days	844 (25.3)	210 (30.6)	
8 days–< 1 month	361 (10.8)	81 (11.8)	
1–2.9 months	1203 (36.1)	234 (34.1)	
≥ 3 months	924 (27.7)	162 (23.6)	

The 2004 Pelotas Cohort Study (*N* = 3374)

Maximum percentage of unknown observation: 224 (6.6%) for children’s skin colour

*SEP* socioeconomic position

Compared to those followed-up at 6 years, children lost to follow-up presented lower SEP, were born to mothers with lower formal education, had more siblings, and presented higher prevalence of low birth weight. There was no difference in maternal age, number of cigarettes per day at pregnancy, child’s sex and skin colour, and duration of exclusive breastfeeding (Table 1). It is important to highlight that we did not observe any differences in the adherence of dietary intake patterns at 1, 2, and 4 years between children followed at 6 years and those lost to follow-up (data not shown).

Tables 2 and 3 show inconsistent associations between dietary intake patterns at 1, 2, and 4 years and *z*-scores of BMI and *corpulence* after adjustment for potential confounders included in the model. *Milks, staple, meat and vegetables, treats*, and *beverages* at the three age-points were not associated with BMI and *corpulence* at 6 years. Higher scores on *snacks* at 4 years independently predicted lower BMI *z*-score at 6 years (*β* = − 0.17; 95% CI − 0.30 to − 0.03). Higher scores on *snacks* also predicted lower *corpulence* at 6 years, but results for this outcome were not significant.

**Table 2:** Adjusted linear regression model between dietary intake patterns at 1, 2, and 4 years and BMI *z*-score at 6 years

	1 year		2 years		4 years	
	*β* (CI 95%)^a^	*p* trend	*β* (CI 95%)^b^	*p* trend	*β* (CI 95%)^c^	*p* trend
Milks						
Low intake (first tertile)	0.00	0.205	0.00	0.451	0.00	0.650
Moderate intake (second tertile)	0.00 (− 0.13; 0.12)		− 0.03 (− 0.16; 0.09)		0.03 (− 0.10; 0.17)	
High intake (third tertile)	− 0.10 (− 0.23; 0.04)		0.06 (− 0.08; 0.21)		0.03 (− 0.10; 0.16)	
Staple						
Low intake (first tertile)	0.00	0.733	0.00	0.085	0.00	0.671
Moderate intake (second tertile)	− 0.10 (− 0.22; 0.03)		0.09 (− 0.04; 0.22)		− 0.03 (− 0.16; 0.10)	
High intake (third tertile)	− 0.02 (− 0.15; 0.10)		0.11 (− 0.02; 0.24)		− 0.03 (− 0.16; 0.10)	
	Meat and vegetables (1 and 2 years)				Treats (4 years)^a^	
Low intake (first tertile)	0.00	0.292	0.00	0.271	0.00	0.597
Moderate intake (second tertile)	0.04 (− 0.09; 0.16)		0.03 (− 0.10; 0.15)		− 0.08 (− 0.21; 0.05)	
High intake (third tertile)	0.07 (− 0.06; 0.20)		0.07 (− 0.06; 0.20)		− 0.03 (− 0.16; 0.09)	
Beverages						
Low intake (first tertile)	0.00	0.332	0.00	0.306	0.00	0.846
Moderate intake (second tertile)	0.01 (− 0.11; 0.13)		− 0.03 (− 0.16; 0.09)		0.05 (− 0.07; 0.18)	
High intake (third tertile)	0.06 (− 0.06; 0.19)		− 0.07 (− 0.19; 0.06)		− 0.01 (− 0.15; 0.12)	
Snacks						
Low intake (first tertile)	0.00	0.112	0.00	0.223	0.00	0.019
Moderate intake (second tertile)	− 0.01 (− 0.14; 0.11)		− 0.11 (− 0.24; 0.02)		− 0.07 (− 0.20; 0.07)	
High intake (third tertile)	− 0.10 (− 0.23; 0.02)		− 0.08 (− 0.22; 0.05)		− 0.17 (− 0.30; − 0.03)	

The 2004 Pelotas Birth Cohort Study, Brazil

^a^Adjusted for: socioeconomic position, number of children at the time of birth, maternal age at birth, maternal education, smoking during pregnancy, child’s sex and skin colour, birth weight, exclusive breastfeeding duration, and number of meals per day

^b^Adjusted for: socioeconomic position, number of children at the time of birth, maternal age at birth, maternal education, smoking during pregnancy, child’s sex and skin colour, birth weight, exclusive breastfeeding duration, number of meals per day, and similar-labeled component at 1 year

^c^Adjusted for: socioeconomic position, number of children at the time of birth, maternal age at birth, maternal education, smoking during pregnancy, child’s sex and skin colour, birth weight, exclusive breastfeeding duration, number of meals per day, and similar-labeled component at 1 and 2 years

**Table 3:** Adjusted linear regression model between dietary intake patterns at 1, 2 and 4 years and *corpulence z*-score at 6 years

	1 year		2 years		4 years	
	*β* (CI 95%)^a^	*p* trend	*β* (CI 95%)^b^	*p* trend	*β* (CI 95%)^c^	*p* trend
Milks						
Low intake (first tertile)	0.00	0.444	0.00	0.832	0.00	0.286
Moderate intake (second tertile)	− 0.02 (− 0.12; 0.07)		− 0.02 (− 0.11; 0.07)		0.04 (− 0.06; 0.13)	
High intake (third tertile)	− 0.04 (− 0.14; 0.06)		0.01 (− 0.09; 0.12)		0.05 (− 0.04; 0.15)	
Staple						
Low intake (first tertile)	0.00	0.245	0.00	0.059	0.00	0.404
Moderate intake (second tertile)	− 0.10 (− 0.20; − 0.01)		0.04 (− 0.05; 0.13)		− 0.02 (− 0.08; 0.11)	
High intake (third tertile)	− 0.06 (− 0.15; 0.04)		0.09 (− 0.01; 0.18)		0.04 (− 0.06; 0.14)	
	Meat and vegetables (1 and 2 years)				Treats^a^	
Low intake (first tertile)	0.00	0.090	0.00	0.126	0.00	0.669
Moderate intake (second tertile)	0.05 (− 0.04; 0.14)		0.03 (− 0.06; 0.12)		0.00 (− 0.10; 0.09)	
High intake (third tertile)	0.08 (− 0.01; 0.18)		0.08 (− 0.02; 0.17)		− 0.02 (− 0.11; 0.07)	
Beverages						
Low intake (first tertile)	0.00	0.489	0.00	0.539	0.00	0.537
Moderate intake (second tertile)	0.04 (− 0.05; 0.13)		− 0.06 (− 0.15; 0.04)		0.03 (− 0.06; 0.13)	
High intake (third tertile)	0.03 (− 0.06; 0.12)		− 0.03 (− 0.13; 0.06)		0.03 (− 0.07; 0.13)	
Snacks						
Low intake (first tertile)	0.00	0.046	0.00	0.306	0.00	0.077
Moderate intake (second tertile)	0.02 (− 0.07; 0.11)		− 0.06 (− 0.15; 0.04)		− 0.07 (− 0.16; 0.03)	
High intake (third tertile)	− 0.09 (− 0.19; 0.00)		− 0.05 (− 0.15; 0.05)		− 0.09 (− 0.19; 0.01)	

The 2004 Pelotas Birth Cohort Study, Brazil

^a^Adjusted for: socioeconomic position, number of children at the time of birth, maternal age at birth, maternal education, smoking during pregnancy, child’s sex and skin colour, birth weight, exclusive breastfeeding duration, and number of meals per day

^b^Adjusted for: socioeconomic position, number of children at the time of birth, maternal age at birth, maternal education, smoking during pregnancy, child’s sex and skin colour, birth weight, exclusive breastfeeding duration, number of meals per day, and similar-labeled component at 1 year

^c^Adjusted for: socioeconomic position, number of children at the time of birth, maternal age at birth, maternal education, smoking during pregnancy, child’s sex and skin colour, birth weight, exclusive breastfeeding duration, number of meals per day, and similar-labeled component at 1 and 2 years

Associations between dietary intake patterns and measures of regional body fatness were also inconsistent after adjustment for confounders. Trunk fat mass percentage was only associated with *staple* intake at 2 years: high score on *staple* at 2 years predicted higher trunk fat mass (*β* = 0.10; 95% CI 0.01–0.19) (Table 4). Gynoid fat mass percentage was the most associated outcome with dietary intake patterns. High scores of *milks* at age of 1 were positively associated with *z*-scores of gynoid fat mass percentage (*β* = 0.16; 95% CI 0.06–0.27), while high scores on *staple* at 2 years were negatively associated with gynoid fat mass percentage at 6 years (*β* = − 0.11; 95% CI − 0.20 to − 0.02). Finally, scoring high on *snacks* at 4 years predicted higher gynoid fat mass percentage *z*-score at age of 6 years (*β* = 0.17; 95% CI 0.07–0.26), independent of the potential confounders included in the models (Table 5).

**Table 4:** Adjusted linear regression model between dietary intake patterns at 1, 2, and 4 years and trunk fat mass percentage *z*-score at 6 years

	1 year		2 years		4 years	
	*β* (CI 95%)^a^	*p* trend	*β* (CI 95%)^b^	*p* trend	*β* (CI 95%)^c^	*p* trend
Milks						
Low intake (first tertile)	0.00	0.084	0.00	0.883	0.00	0.763
Moderate intake (second tertile)	− 0.06 (− 0.15; 0.03)		− 0.02 (− 0.11; 0.07)		− 0.02 (− 0.11; 0.07)	
High intake (third tertile)	− 0.08 (− 0.18; 0.02)		− 0.01 (− 0.11; 0.10)		0.01 (− 0.08; 0.10)	
Staple						
Low intake (first tertile)	0.00	0.168	0.00	0.027	0.00	0.299
Moderate intake (second tertile)	− 0.09 (− 0.18; 0.00)		0.07 (− 0.02; 0.16)		− 0.03 (− 0.12; 0.06)	
High intake (third tertile)	− 0.06 (− 0.15; 0.03)		0.10 (0.01; 0.19)		− 0.05 (− 0.14; 0.04)	
	Meat and vegetables (1 and 2 years)				Treats^a^	
Low intake (first tertile)	0.00	0.962	0.00	0.868	0.00	0.859
Moderate intake (second tertile)	0.01 (− 0.08; 0.09)		0.03 (− 0.06; 0.12)		− 0.07 (− 0.15; 0.02)	
High intake (third tertile)	− 0.00 (− 0.09; 0.09)		0.01 (− 0.08; 0.10)		− 0.01 (− 0.10; 0.08)	
Beverages						
Low intake (first tertile)	0.00	0.088	0.00	0.372	0.00	0.954
Moderate intake (second tertile)	0.06 (− 0.03; 0.15)		− 0.02 (− 0.11; 0.07)		− 0.01 (− 0.10; 0.08)	
High intake (third tertile)	0.08 (− 0.01; 0.16)		0.04 (− 0.05; 0.13)		0.00 (− 0.09; 0.09)	
Snacks						
Low intake (first tertile)	0.00	0.158	0.00	0.310	0.00	0.062
Moderate intake (second tertile)	− 0.04 (− 0.12; 0.05)		− 0.06 (− 0.15; 0.04)		− 0.09 (− 0.18; 0.00)	
High intake (third tertile)	− 0.06 (− 0.15; 0.02)		− 0.05 (− 0.14; 0.05)		− 0.09 (− 0.18; 0.01)	

The 2004 Pelotas Birth Cohort Study, Brazil

^a^Adjusted for: socioeconomic position, number of children at the time of birth, maternal age at birth, maternal education, smoking during pregnancy, child’s sex and skin colour, birth weight, exclusive breastfeeding duration, and number of meals per day

^b^Adjusted for: socioeconomic position, number of children at the time of birth, maternal age at birth, maternal education, smoking during pregnancy, child’s sex and skin colour, birth weight, exclusive breastfeeding duration, number of meals per day, and similar-labeled component at 1 year

^c^Adjusted for: socioeconomic position, number of children at the time of birth, maternal age at birth, maternal education, smoking during pregnancy, child’s sex and skin colour, birth weight, exclusive breastfeeding duration, number of meals per day, and similar-labeled component at 1 and 2 years

**Table 5:** Adjusted linear regression model between dietary intake patterns at 1, 2, and 4 years and gynoid fat mass percentage *z*-score at 6 years

	1 year		2 years		4 years	
	*β* (CI 95%)^a^	*p* trend	*β* (CI 95%)^b^	*p* trend	*β* (CI 95%)^c^	*p* trend
Milks						
Low intake (first tertile)	0.00	0.001	0.00	0.512	0.00	0.172
Moderate intake (second tertile)	0.06 (− 0.03; 0.14)		0.00 (− 0.09; 0.09)		− 0.03 (− 0.13; 0.06)	
High intake (third tertile)	0.16 (0.06; 0.27)		− 0.04 (− 0.14; 0.06)		− 0.06 (− 0.16; 0.03)	
Staple						
Low intake (first tertile)	0.00	0.340	0.00	0.020	0.00	0.388
Moderate intake (second tertile)	0.07 (− 0.01; 0.16)		− 0.08 (− 0.17; 0.01)		0.03 (− 0.06; 0.12)	
High intake (third tertile)	0.04 (− 0.05; 0.13)		− 0.11 (− 0.20; − 0.02)		0.04 (− 0.05; 0.13)	
	Meat and vegetables (1 and 2 years)				Treats^a^	
Low intake (first tertile)	0.00	0.559	0.00	0.708	0.00	0.811
Moderate intake (second tertile)	0.01 (− 0.08; 0.09)		− 0.05 (− 0.14; 0.04)		0.04 (− 0.05; 0.13)	
High intake (third tertile)	− 0.03 (− 0.12; 0.06)		− 0.02 (− 0.11; 0.07)		0.01 (− 0.08; 0.10)	
Beverages						
Low intake (first tertile)	0.00	0.179	0.00	0.890	0.00	0.440
Moderate intake (second tertile)	− 0.03 (− 0.11; 0.06)		0.02 (− 0.07; 0.11)		0.01 (− 0.08; 0.10)	
High intake (third tertile)	− 0.06 (− 0.15; 0.03)		− 0.01 (− 0.10; 0.08)		0.04 (− 0.05; 0.13)	
Snacks						
Low intake (first tertile)	0.00	0.138	0.00	0.053	0.00	0.001
Moderate intake (second tertile)	− 0.01 (− 0.10; 0.08)		0.05 (− 0.04; 0.14)		0.05 (− 0.04; 0.14)	
High intake (third tertile)	0.07 (− 0.02; 0.15)		0.09 (0.00; 0.18)		0.17 (0.07; 0.26)	

The 2004 Pelotas Birth Cohort Study, Brazil

^a^Adjusted for: socioeconomic position, number of children at the time of birth, maternal age at birth, maternal education, smoking during pregnancy, child’s sex and skin colour, birth weight, exclusive breastfeeding duration, and number of meals per day

^b^Adjusted for: socioeconomic position, number of children at the time of birth, maternal age at birth, maternal education, smoking during pregnancy, child’s sex and skin colour, birth weight, exclusive breastfeeding duration, number of meals per day, and similar-labeled component at 1 year

^c^Adjusted for: socioeconomic position, number of children at the time of birth, maternal age at birth, maternal education, smoking during pregnancy, child’s sex and skin colour, birth weight, exclusive breastfeeding duration, number of meals per day, and similar-labeled component at 1 and 2 years

## Discussion

Our study in this Brazilian population-based sample showed that there were no consistent associations between dietary intake patterns from 1 to 4 years and BMI and body shape at age of 6 years. The previous studies in different settings have demonstrated no consistent associations between dietary intake patterns and BMI and adiposity in children and adolescents [14, 15, 28, 29]. Nonetheless, other investigations have suggested that association between dietary intake and BMI in children would be better explained by the amount of fat-free mass than fat mass [30, 31]. To test this hypothesis, we performed extra analysis using fat-free mass index from DXA as the outcome, adjusting for the same potential confounders used for the other outcomes. As we observed in supplementary table 5, there were no associations between dietary intake patterns at 1, 2, and 4 years and fat-free mass index at 6 years.

Even with the lack of significance in the majority of associations tested here, our results showed that the dietary component labeled *staple* at 2 years, and the other one labeled *snacks* at 4 years were the only ones which remained associated with more than one outcome, despite the effect sizes seen had been small for these associations. High score on *staple*, characterized by high adherence to rice and beans, the ubiquitous combination in Brazilian diet, was associated with higher trunk fat mass and with lower gynoid fat mass at 6 years. Furthermore, high score on *snacks*, characterized by high adherence to coffee, bread and cookies, appeared to be associated with lower BMI, but higher gynoid fat mass percentage at 6 years.

Interpretation of results for BMI *z*-score is easier, as BMI is a valuable adiposity marker for diseases and mortality risks, and is widely used in clinical and epidemiological studies [32, 33]. Greater caution is needed when interpreting the results for the novel body shape component “*corpulence*”, since its utility in predicting future adverse outcomes is not known. However, as *corpulence* is very highly correlated with BMI and other adiposity measures (*r* > 0.9) [20], and results for this component followed a similar trend that those for BMI, we can presume that interpretation for this body shape component goes in the same direction as for BMI.

In addition, we conducted further analyses using regional measures of body shape from DXA (trunk and gynoid fat mass percentage) to help in interpreting results for body shape. In these analyses, we have seen that high score on *snacks* at 4 years predicted higher proportion of gynoid fat mass at 6 years, while high score on *staple* at 2 years predicted higher proportion of trunk fat mass and lower proportion of gynoid fat mass. The previous epidemiological and mechanistic studies have demonstrated that higher proportion of fat mass in central area increases risk of adverse outcomes, while peripheral fat is beneficial for metabolic disorders [34–38], and our results may be indicating that lower adherence to rice and beans at 2 years, and high adherence to coffee, bread, and cookies at 4 years could be beneficial to body shape at 6 years, decreasing the overall body size, and improving the distribution of body fat.

However, what might be the reason for high adherence to *snacks* at 4 years promotes lower BMI and better body shape? Higher scores on *snacks* might be associated with some factors, such as age of introduction of foods, food availability at household, or quality of diet, for example, which may interfere on BMI and body shape of these children. However, to support this hypothesis, additional studies focusing not solely on dietary patterns but also on other aspects of feeding habits are needed to better understand the role of early feeding habits, especially of *snacks* adherence, on BMI and distribution of body fat in childhood.

Nevertheless, we cannot reject the hypothesis of residual confounding by other socioeconomic or demographic factors not included in the adjusted model, since the observed effect sizes of the associations were small (at best, *β* = 0.20 *z*-score), despite *p* values < 0.05. Moreover, we also evidenced a considerable reduction in estimated effects for all outcomes included in our analyses (> 40%) after adjustment for potential confounders. The published reports from this cohort and other studies have already showed that dietary intake patterns are highly associated with socioeconomic and demographic characteristics [21, 28, 39]. Finally, since many associations were tested here, we also must be aware about the multiple testing problem, which might increase the probability of type I error in the associations investigated [40]. Nonetheless, the good power of our sample helps to address this issue.

Another notable finding of our study was the association between the component labeled *milks* at 1 year and body shape measures from DXA. High adherence to *milks* at 1 year (characterized by high adherence to breast milk) predicted higher gynoid fat mass at 6 years. This result may indicate a possible benefit of breast milk for body shape. The role of breastfeeding on obesity status in childhood remains debated [41]. Unfortunately, here, we did not address the effects of breastfeeding duration on body shape, and future investigations would be interesting to look at the role of exclusive and total breastfeeding duration on obesity status and body shape as well as the mechanism involved in this relationship.

A strength of our study is the repeated assessment of food intake, allowing specific descriptions of dietary patterns during infancy and early childhood. The high retention rates (above 90%) in all follow-ups as well as regular data collection are important to minimize biases. Outcome assessment is a further strength, as our study did not focus solely on BMI, but used a novel consideration of body size and body shape that reflects adiposity and fat distribution added by regional fat mass measures from DXA.

Limitations include the assessment of food intake by 24-h recall reported by mothers, which may not reflect children’s regular feeding habits. This indirect measure can result in measurement error, since mothers can overestimate total energy intake of children [42]. In addition, the questionnaires used in 1-, 2-, and 4-year-old follow-ups did not allow us to quantify food amounts and the dietary intake patterns described in these three age-points do not reflect overall dietary quality. Finally, we were not able to employ the full strength of longitudinal analyses in our study, since we have run separate linear regression models by each age-point. We performed analyses separately by each age-point due to the variation in food item loadings of components with the same label as well as the weak inter-correlations between components.

In conclusion, our study adds evidence that the consumption of dietary patterns in the early life presents inconsistent association with BMI and body shape at 6 years, as all the effects seen here were attenuated by adjustment for socioeconomic and demographic characteristics. However, lower adherence to *staple* at 2 years and high adherence to *snacks* at 4 years may promote a better body shape, with lower BMI and central fat and higher proportion of gynoid fat mass.

## Electronic supplementary material

Below is the link to the electronic supplementary material.

Supplementary material 1 (DOCX 13 KB)

Supplementary material 2 (DOCX 14 KB)

Supplementary material 3 (DOCX 14 KB)

Supplementary material 4 (DOCX 14 KB)

Supplementary material 5 (DOCX 14 KB)
