# Supplementary material for: Effects of dietary intake patterns from 1 to 4 years on BMI z-score and body shape at age of 6 years: a prospective birth cohort study from Brazil
Source: Eur J Nutr. 2018 May 17;58(4):1723–34. doi: 10.1007/s00394-018-1720-3 (PMC6562047; doi:10.1007/s00394-018-1720-3)
Supplement: Supplementary file 1 — Supplementary material 1 (DOCX 13 KB) [file 394_2018_1720_MOESM1_ESM.docx]

**Supplementary table 1.** Dietary components of dietary intake patterns at 1, 2 and 4 years of children from the 2004 Pelotas birth cohort.

| **Dietary components** | **Food item** | **Loading** | **% Variance** |
| --- | --- | --- | --- |
| **Dietary intake patterns at 1 year** | | | |
| 1. Milks | Breast milk  Cow’s milk | 0.68  -0.70 | 11.2 |
| 2. Staple | Rice  Beans  Pasta | 0.65  0.55  -0.40 | 11.1 |
| 3. Meat and vegetables | Meat  Vegetable/legumes  Potato/cassava | 0.68  0.50  0.36 | 9.1 |
| 4. Beverages | Juice  Water/tea | 0.66  -0.72 | 8.6 |
| 5. Snacks | Coffee  Bread/cookies  Fruits | 0.53  0.63  -0.38 | 8.4 |
| **Dietary intake patterns at 2 years** | | | |
| 1. Milks | Breast milk  Cow’s milk | 0.65  -0.69 | 9.5 |
| 2. Staple | Rice  Beans  Pasta | 0.67  0.61  -0.38 | 10.8 |
| 3. Meat and vegetables | Meat  Vegetable/legumes  Potato/cassava  Fruits | 0.56  0.54  0.38  0.46 | 8.0 |
| 4. Beverages | Juice  Water/tea | 0.68  -0.71 | 8.4 |
| 5. Snacks | Coffee  Bread/cookies  Yogurt | 0.58  0.58  -0.45 | 9.1 |
| **Dietary intake patterns at 4 years** | | | |
| 1. Milks | Cow’s milk  Chocolate Milk | 0.64  0.62 | 9.2 |
| 2. Staple | Rice  Beans  Meat | 0.62  0.50  0.42 | 9.4 |
| 3. Beverages | Juice  Soft drinks | 0.71  -0.52 | 7.2 |
| 4. Snacks | Coffee  Bread/cookies  Water/tea  Yogurt  Soft drinks | 0.46  0.35  0.34  -0.42  -0.39 | 7.6 |
| 5. Treats | Chips  Sweets  Chocolate | 0.58  0.57  0.43 | 7.1 |
