# Supplementary material for: Effects of dietary intake patterns from 1 to 4 years on BMI z-score and body shape at age of 6 years: a prospective birth cohort study from Brazil
Source: Eur J Nutr. 2018 May 17;58(4):1723–34. doi: 10.1007/s00394-018-1720-3 (PMC6562047; doi:10.1007/s00394-018-1720-3)
Supplement: Supplementary file 2 — Supplementary material 2 (DOCX 14 KB) [file 394_2018_1720_MOESM2_ESM.docx]

**Supplementary table 2.** Crude linear regression model between dietary intake patterns at 1, 2 and 4 years and z-scores of BMI and *corpulence* at 6y. The 2004 Pelotas Birth Cohort Study, Brazil.

|  | **1 year** | | **2 years** | | **4 years** | | | |
| --- | --- | --- | --- | --- | --- | --- | --- | --- |
|  | **BMI**  **β (CI 95%)** | **Corpulence**  **β (CI 95%)** | **BMI**  **β (CI 95%)** | **Corpulence**  **β (CI 95%)** | **BMI**  **β (CI 95%)** | **Corpulence**  **β (CI 95%)** | | |
| **Milks** | | | | | | | | |
| Low intake | 0.00 | 0.00 | 0.00 | 0.00 | 0.00 | | 0.00 | |
| Moderate intake | 0.03 (-0.09; 0.16) | 0.01 (-0.08; 0.10) | 0.02 (-0.10; 0.14) | 0.03 (-0.07; 0.12) | 0.15 (0.03; 0.27) | | 0.13 (0.04; 0.22) | |
| High intake | -0.13 (-0.25; -0.01) | -0.07 (-0.16; 0.02) | -0.01 (-0.13; 0.11) | -0.02 (-0.11; 0.07) | 0.21 (0.09; 0.33) | | 0.20 (0.10; 0.29) | |
| **Staple** | | | | | | | | |
| Low intake | 0.00 | 0.00 | 0.00 | 0.00 | 0.00 | | 0.00 | |
| Moderate intake | -0.13 (-0.25; -0.01) | -0.14 (-0.23; -0.05) | -0.02 (-0.14; 0.10) | -0.05 (-0.14; 0.05) | -0.11 (-0.23; 0.01) | | -0.06 (-0.15; 0.04) | |
| High intake | -0.18 (-0.30; -0.06) | -0.18 (-0.27; -0.09) | -0.04 (-0.16; 0.08) | -0.06 (-0.15; 0.03) | -0.16 (-0.28; -0.04) | | -0.07 (-0.16; 0.03) | |
|  | **Meat and vegetables (1 & 2y)** | | | | **Treats (4y)** | | | |
| Low intake | 0.00 | 0.00 | 0.00 | 0.00 | 0.00 | | 0.00 | |
| Moderate intake | 0.14 (0.02; 0.26) | 0.16 (0.07; 0.25) | 0.02 (-0.10; 0.15) | 0.06 (-0.03; 0.15) | -0.12 (-0.24; 0.00) | | -0.06 (-0.15; 0.03) | |
| High intake | 0.29 (0.16; 0.41) | 0.28 (0.19; 0.37) | 0.15 (0.03; 0.28) | 0.16 (0.07; 0.25) | -0.14 (-0.26; -0.02) | | -0.12 (-0.21; -0.02) | |
| **Beverages** | | | | | | | | |
| Low intake | 0.00 | 0.00 | 0.00 | 0.00 | 0.00 | | 0.00 | |
| Moderate intake | -0.02 (-0.14; 0.11) | 0.02 (-0.07; 0.11) | -0.04 (-0.16; 0.09) | -0.05 (-0.14; 0.04) | 0.05 (-0.07; 0.18) | | 0.07 (-0.02; 0.16) | |
| High intake | 0.12 (-0.01; 0.24) | 0.09 (0.00; 0.18) | 0.00 (-0.12; 0.12) | 0.04 (-0.05; 0.13) | 0.04 (-0.08; 0.16) | | 0.07 (-0.02; 0.16) | |
| **Snacks** | | | | | | | | |
| Low intake | 0.00 | 0.00 | 0.00 | 0.00 | 0.00 | | | 0.00 |
| Moderate intake | -0.09 (-0.21; 0.03) | -0.07 (-0.16; 0.03) | -0.19 (-0.31; -0.07) | -0.12 (-0.22; -0.03) | -0.11 (-0.23; 0.02) | | | -0.09 (-0.18; 0.00) |
| High intake | -0.26 (-0.38; -0.14) | -0.23 (-0.33; -0.14) | -0.33 (-0.45; -0.20) | -0.27 (-0.36; -0.18) | -0.42 (-0.54; -0.30) | | | -0.32 (-0.41; -0.23) |
