# Supplementary material for: Effects of dietary intake patterns from 1 to 4 years on BMI z-score and body shape at age of 6 years: a prospective birth cohort study from Brazil
Source: Eur J Nutr. 2018 May 17;58(4):1723–34. doi: 10.1007/s00394-018-1720-3 (PMC6562047; doi:10.1007/s00394-018-1720-3)
Supplement: Supplementary file 3 — Supplementary material 3 (DOCX 14 KB) [file 394_2018_1720_MOESM3_ESM.docx]

**Supplementary table 3.** Crude linear regression model between dietary intake patterns at 1, 2 and 4 years and z-scores of trunk and gynoid fat mass at 6y. The 2004 Pelotas Birth Cohort Study, Brazil.

|  | **1 year** | | **2 years** | | **4 years** | | | |
| --- | --- | --- | --- | --- | --- | --- | --- | --- |
|  | **Trunk fat mass**  **β (CI 95%)** | **Gynoid fat mass**  **β (CI 95%)** | **Trunk fat mass**  **β (CI 95%)** | **Gynoid fat mass**  **β (CI 95%)** | **Trunk fat mass**  **β (CI 95%)** | **Gynoid fat mass**  **β (CI 95%)** | | |
| **Milks** | | | | | | | | |
| Low intake | 0.00 | 0.00 | 0.00 | 0.00 | 0.00 | | 0.00 | |
| Moderate intake | -0.03 (-0.12; 0.05) | 0.01 (-0.08; 0.09) | 0.01 (-0.07; 0.10) | -0.04 (-0.13; 0.04) | 0.06 (-0.03; 0.14) | | -0.10 (-0.19; -0.02) | |
| High intake | -0.07 (-0.15; 0.02) | 0.14 (0.05; 0.22) | -0.01 (-0.09; 0.08) | 0.01 (-0.07; 0.10) | 0.11 (0.03; 0.20) | | -0.19 (-0.28; -0.11) | |
| **Staple** | | | | | | | | |
| Low intake | 0.00 | 0.00 | 0.00 | 0.00 | 0.00 | | 0.00 | |
| Moderate intake | -0.09 (-0.18; -0.01) | 0.10 (0.01; 0.18) | 0.01 (-0.07; 0.10) | -0.03 (-0.12; 0.05) | -0.04 (-0.12; 0.05) | | 0.09 (0.00; 0.17) | |
| High intake | -0.10 (-0.19; -0.02) | 0.12 (0.04; 0.20) | 0.03 (-0.06; 0.11) | 0.00 (-0.09; 0.08) | -0.10 (-0.19; -0.02) | | 0.13 (0.05; 0.22) | |
|  | **Meat and vegetables (1 & 2y)** | | | | **Treats (4y)** | | | |
| Low intake | 0.00 | 0.00 | 0.00 | 0.00 | 0.00 | | 0.00 | |
| Moderate intake | 0.05 (-0.03; 0.14) | -0.07 (-0.16; 0.01) | 0.06 (-0.02; 0.15) | -0.05 (-0.14; 0.03) | -0.09 (-0.18; -0.01) | | 0.05 (-0.03; 0.14) | |
| High intake | 0.07 (-0.02; 0.15) | -0.16 (-0.24; -0.07) | 0.07 (-0.02; 0.15) | -0.08 (-0.16; 0.01) | -0.03 (-0.12; 0.05) | | 0.04 (-0.04; 0.13) | |
| **Beverages** | | | | | | | | |
| Low intake | 0.00 | 0.00 | 0.00 | 0.00 | 0.00 | | 0.00 | |
| Moderate intake | 0.02 (-0.06; 0.11) | 0.00 (-0.08; 0.09) | -0.06 (-0.14; 0.03) | 0.04 (-0.05; 0.12) | -0.02 (-0.11; 0.06) | | 0.04 (-0.05; 0.12) | |
| High intake | 0.08 (0.00; 0.17) | -0.08 (-0.16; 0.01) | 0.03 (-0.06; 0.11) | -0.02 (-0.10; 0.07) | 0.00 (-0.09; 0.08) | | 0.05 (-0.04;0.13) | |
| **Snacks** | | | | | | | | |
| Low intake | 0.00 | 0.00 | 0.00 | 0.00 | 0.00 | | | 0.00 |
| Moderate intake | -0.04 (-0.13; 0.04) | 0.03 (-0.06; 0.11) | -0.09 (-0.18; -0.01) | 0.10 (0.02; 0.18) | -0.11 (-0.19; -0.02) | | | 0.05 (-0.03; 0.14) |
| High intake | -0.11 (-0.20; -0.03) | 0.15 (0.07; 0.24) | -0.14 (-0.23; -0.06) | 0.23 (0.14; 0.31) | -0.19 (-0.27; -0.10) | | | 0.31 (0.22; 0.39) |
