# Supplementary material for: Effects of dietary intake patterns from 1 to 4 years on BMI z-score and body shape at age of 6 years: a prospective birth cohort study from Brazil
Source: Eur J Nutr. 2018 May 17;58(4):1723–34. doi: 10.1007/s00394-018-1720-3 (PMC6562047; doi:10.1007/s00394-018-1720-3)
Supplement: Supplementary file 4 — Supplementary material 4 (DOCX 14 KB) [file 394_2018_1720_MOESM4_ESM.docx]

**Supplementary table 4.** Number of meals per day at 1, 2 and 4 years according to adherence of each dietary intake component at the three age-points. The 2004 Pelotas Birth Cohort Study, Brazil.

| **Adherence** | **1 year** | **2 years** | **4 years** |  |  |
| --- | --- | --- | --- | --- | --- |
|  | **Mean (sd)** | **Mean (sd)** | **Mean (sd)** |  |  |
|  | **Milks** | | | |  |
| *p-value* | *<0.001* | *<0.001* | *<0.001* | |  |
| Low (1st tertile) | 6.4 (0.6) | 6.1 (0.7) | 4.9 (0.9) | |  |
| Moderate (2^nd^ tertile) | 5.9 (0.7) | 5.7 (0.8) | 5.2 (0.8) | |  |
| High (3^rd^ tertile) | 6.6 (0.6) | 6.0 (1.0) | 5.4 (0.8) | |  |
|  | **Staple** | | | |  |
| *p-value* | *0.002* | *<0.001* | *<0.001* | |  |
| Low (1st tertile) | 6.2 (0.8) | 5.8 (0.9) | 5.0 (0.9) | |  |
| Moderate (2^nd^ tertile) | 6.3 (0.7) | 5.9 (0.9) | 5.2 (0.9) | |  |
| High (3^rd^ tertile) | 6.4 (0.7) | 6.0 (0.8) | 5.3 (0.8) | |  |
|  | **Meat and vegetables (1 & 2y)** | | **Treats (4y)** | |  |
| *p-value* | *<0.001* | *<0.001* | *0.799* | |  |
| Low (1st tertile) | 6.2 (0.8) | 5.7 (0.9) | 5.2 (0.9) | |  |
| Moderate (2^nd^ tertile) | 6.3 (0.7) | 5.9 (0.9) | 5.2 (0.9) | |  |
| High (3^rd^ tertile) | 6.4 (0.7) | 6.0 (0.8) | 5.2 (0.9) | |  |
|  | **Beverages** | | | |  |
| *p-value* | *0.188* | *0.035* | *<0.001* | |  |
| Low (1st tertile) | 6.3 (0.7) | 5.9 (0.8) | 5.0 (0.9) | |  |
| Moderate (2^nd^ tertile) | 6.3 (0.8) | 5.9 (0.9) | 5.2 (0.9) | |  |
| High (3^rd^ tertile) | 6.3 (0.7) | 5.9 (0.8) | 5.3 (0.8) | |  |
|  | **Snacks** | | | | |
| *p-value* | *<0.001* | *<0.001* | *<0.001* | | |
| Low (1st tertile) | 6.3 (0.7) | 5.8 (0.8) | 5.3 (0.9) | | |
| Moderate (2^nd^ tertile) | 6.3 (0.8) | 5.9 (0.8) | 5.1 (0.9) | | |
| High (3^rd^ tertile) | 6.4 (0.7) | 6.0 (0.9) | 5.1 (0.9) | | |

sd = standard deviation
