# Supplementary material for: Effects of dietary intake patterns from 1 to 4 years on BMI z-score and body shape at age of 6 years: a prospective birth cohort study from Brazil
Source: Eur J Nutr. 2018 May 17;58(4):1723–34. doi: 10.1007/s00394-018-1720-3 (PMC6562047; doi:10.1007/s00394-018-1720-3)
Supplement: Supplementary file 5 — Supplementary material 5 (DOCX 14 KB) [file 394_2018_1720_MOESM5_ESM.docx]

**Supplementary table 5.** Adjusted linear regression model between dietary intake patterns at 1, 2 and 4 years and fat-free mass index at 6y. The 2004 Pelotas Birth Cohort Study, Brazil.

|  | **1 year** | | **2 years** | | **4 years** | | | |
| --- | --- | --- | --- | --- | --- | --- | --- | --- |
|  | **β (CI 95%)*** | **p-trend** | **β (CI 95%)†** | **p-trend** | **β (CI 95%)††** | **p-trend** | | |
| **Milks** | | | | | | | | |
| Low intake (1st tertile) | 0.00 | *0.584* | 0.00 | *0.582* | 0.00 | | *0.189* | |
| Moderate intake (2^nd^ tertile) | 0.04 (-0.04;0.12) |  | -0.01 (-0.09; 0.07) |  | -0.04 (-0.13; 0.05) | |  | |
| High intake (3^rd^ tertile) | 0.02 (-0.07;0.11) |  | 0.03 (-0.07; 0.13) |  | -0.06 (-0.15; 0.03) | |  | |
| **Staple** | | | | | | | | |
| Low intake (1st tertile) | 0.00 | *0.100* | 0.00 | *0.123* | 0.00 | | *0.511* | |
| Moderate intake (2^nd^ tertile) | -0.03 (-0.11; 0.05) |  | 0.04 (-0.04; 0.13) |  | 0.02 (-0.06; 0.11) | |  | |
| High intake (3^rd^ tertile) | 0.07 (-0.01; 0.15) |  | 0.07 (-0.02; 0.15) |  | 0.03 (-0.06; 0.11) | |  | |
|  | **Meat and vegetables (1 & 2y)** | | | | **Treats (4y)*** | | | |
| Low intake (1st tertile) | 0.00 | *0.557* | 0.00 | *0.426* | 0.00 | | *0.075* | |
| Moderate intake (2^nd^ tertile) | 0.05 (-0.04; 0.13) |  | 0.01 (-0.07; 0.09) |  | 0.00 (-0.08; 0.08) | |  | |
| High intake (3^rd^ tertile) | 0.03 (-0.06; 0.11) |  | 0.03 (-0.05; 0.12) |  | 0.08 (-0.01; 0.16) | |  | |
| **Beverages** | | | | | | | | |
| Low intake (1st tertile) | 0.00 | *0.556* | 0.00 | *0.490* | 0.00 | | *0.163* | |
| Moderate intake (2^nd^ tertile) | 0.03 (-0.06; 0.11) |  | -0.02 (-0.11; 0.06) |  | 0.06 (-0.02; 0.14) | |  | |
| High intake (3^rd^ tertile) | 0.02 (-0.06; 0.11) |  | -0.03 (-0.11; 0.05) |  | -0.06 (-0.15; 0.02) | |  | |
| **Snacks** | | | | | | | | |
| Low intake (1st tertile) | 0.00 | *0.820* | 0.00 | *0.814* | 0.00 | | | *0.742* |
| Moderate intake (2^nd^ tertile) | 0.02 (-0.07; 0.10) |  | -0.05 (-0.13; 0.04) |  | 0.00 (-0.09; 0.09) | | |  |
| High intake (3^rd^ tertile) | 0.01 (-0.07; 0.09) |  | 0.01 (-0.08; 0.10) |  | 0.02 (-0.08; 0.11) | | |  |

* Adjusted for: socioeconomic position, number of children at the time of birth, maternal age at birth, maternal education, smoking during pregnancy, child’s sex and skin colour, birth weight, exclusive breastfeeding duration, and number of meals per day.

† Adjusted for: socioeconomic position, number of children at the time of birth, maternal age at birth, maternal education, smoking during pregnancy, child’s sex and skin colour, birth weight, exclusive breastfeeding duration, number of meals per day, and similar-labeled component at 1 year.

†† Adjusted for: socioeconomic position, number of children at the time of birth, maternal age at birth, maternal education, smoking during pregnancy, child’s sex and skin colour, birth weight, exclusive breastfeeding duration, number of meals per day, and similar-labeled component at 1 year and 2 years.
